# Photoemission Spectra from the Extended Koopman’s Theorem, Revisited

**DOI:** 10.3389/fchem.2021.746735

**Published:** 2021-10-08

**Authors:** S. Di Sabatino, J. Koskelo, J. Prodhon, J. A. Berger, M. Caffarel, P. Romaniello

**Affiliations:** ^1^ Laboratoire de Chimie et Physique Quantiques, Université de Toulouse, CNRS, UPS, Toulouse, France; ^2^ Laboratoire de Physique Théorique, Université de Toulouse, CNRS, UPS, Toulouse, France; ^3^ European Theoretical Spectroscopy Facility (ETSF), Toulouse, France

**Keywords:** extended Koopman’s theorem, strong correlation, photoemission, one-body Green’s function, RDMFT, QMC

## Abstract

The Extended Koopman’s Theorem (EKT) provides a straightforward way to compute charged excitations from any level of theory. In this work we make the link with the many-body effective energy theory (MEET) that we derived to calculate the spectral function, which is directly related to photoemission spectra. In particular, we show that at its lowest level of approximation the MEET removal and addition energies correspond to the so-called diagonal approximation of the EKT. Thanks to this link, the EKT and the MEET can benefit from mutual insight. In particular, one can readily extend the EKT to calculate the full spectral function, and choose a more optimal basis set for the MEET by solving the EKT secular equation. We illustrate these findings with the examples of the Hubbard dimer and bulk silicon.

## 1 Introduction

The Extended Koopman’s Theorem (EKT) (Morrell et al., 1975; Smith and Day, 1975) has been derived in quantum chemistry and used within various frameworks, from functional theories based on reduced quantities, such as reduced-density matrix functional theory (Gilbert, 1975) (e.g., Pernal and Cioslowski, 2005; Leiva and Piris, 2005; Piris et al., 2012; Piris et al., 2013) and many-body perturbation theory based on Green’s functions (Hedin, 1965) (e.g., Dahlen and van Leeuwen, 2005; Stan et al., 2006; Stan et al., 2009), to wavefunction-based methods (e.g., Cioslowski et al., 1997; Kent et al., 1998; Bozkaya, 2013; Zheng, 2016; Bozkaya and Ünal, 2018; Pavlyukh, 2019; Lee et al., 2021). The EKT allows one to calculate energies corresponding to charged excitations. Although it can be formulated both for ionization potentials (IPs) and electron affinities (EAs), it has been widely used only for the former, whereas for the latter applications have been limited to the calculation of the lowest EA as the first IP of the (*N*+1)-electron system (in case of finite systems), with *N* the number of electrons in the reference system. There exist hence many benchmarks for the IPs. So far, the method has been mainly used for finite systems. The EKT is known to be in principle (i.e., using exact ingredients, namely the one- and two body density matrices, as we shall see) exact for the first ionization potential (Katriel and Davidson, 1980; Sundholm and Olsen, 1993). In the solid state instead there are only a few applications which only focus on the band structure. It would be desirable to have also the spectral function, i.e., the spectrum of electron addition and removal energies weighted by the Dyson amplitudes, which measure the overlap between the eigenstates of the (*N* + 1) − electron ((*N* − 1) − electron) system and the ground state of the *N*-electron system where an electron has been added (removed). The spectral function is related to photoemission spectroscopy, which gives precious information about the electronic structure and excitations in a system, and, moreover, allows one to study metal-insulator transitions, of paramount importance in condensed matter.

A simple way to calculate the spectral function *A*(*ω*) is through the imaginary part of the one-body Green’s function *G*(*ω*), as $A\left(\omega \right)=\frac{1}{\pi }\text{sgn}\left(\mu -\omega \right)IG\left(\omega \right)$ , where *μ* is the chemical potential. The one-body Green’s function is the fundamental quantity of many-body perturbation theory; it can be obtained from the Dyson equation *G* = *G*_0_ + *G*_0_Σ*G*, in which *G*_0_ and *G* are the noninteracting and interacting Green’s functions, respectively, and Σ the so-called self-energy, which contains all the many-body effects of the system. This latter quantity needs to be approximated in practical calculations. Commonly used approximations, such as the well-known *GW* approximation (Hedin, 1965), cannot capture the Mott physics (Romaniello et al., 2009; Romaniello et al., 2012; Di Sabatino et al., 2015; Di Sabatino et al., 2016; Di Sabatino et al., 2021). Therefore much effort is devoted to develop better approximations to Σ (Springer et al., 1998; Zhukov et al., 2004; Shishkin et al., 2007; Kuneš et al., 2007; Guzzo et al., 2011; Romaniello et al., 2012; Lischner et al., 2013; Stefanucci et al., 2014) or to develop novel ways to determine *G* (Lani et al., 2012; Berger et al., 2014). In this spirit in these last years we have developed the many-body effective energy theory (MEET) (Di Sabatino et al., 2016), in which the spectral function is expressed in terms of density matrices, or, alternatively, in terms of moments of *G*, as reported in Ref. (Di Sabatino et al., 2019). This has allowed us to describe the band gap in several paramagnetic transition-metal oxides (Di Sabatino et al., 2016; Di Sabatino et al., 2019; Di Sabatino et al., 2021), such as NiO, which are considered strongly correlated materials and which are described as metals by static mean-field theories, such as DFT, and by *GW*. This is an important result. However the band gap is hugely overestimated by the MEET within the current low-order approximation in terms of the (approximate) one- and two-body density matrices. Improvements are needed, either by going to higher-order density matrices, which, however, is not guaranteed to converge, or by introducing some sort of screening in the equations. Recently we have obtained promising results for the description of the insulator-to-metal transition of PM FeO under pressure by combining the MEET and the local-density approximation (LDA) (Di Sabatino et al., 2021), and we are currently working on introducing electron-hole screening in the MEET equations. However there is another path which we can explore, and this comes from the relation between the MEET and the EKT. As we will show in the following, within a given basis, the removal and addition energies obtained within the MEET at the lowest-order approximation are equal to the EKT removal and addition energies within the diagonal approximation. In this work we discuss this link and its impact on both theories.

The paper is organized as follows. In *Theory* we give the basic equations of the EKT and the MEET and we make the link between them. The Hubbard dimer and bulk silicon are used to illustrate the difference between the EKT and the MEET (removal and addition) energies in *Mutual Insights and Illustration*. In *Conclusions and Perspectives* we draw our conclusions and perspectives.

## 2 Theory

In this section we briefly review the MEET and EKT methods, and we make the link between the two. We will consider an *N*-electron system governed by the following Hamiltonian in second quantization

$$\hat{H}=\underset{ij}{\sum }h_{ij}{\hat{a}}_{i}^{†}{\hat{a}}_{j}+\frac{1}{2}\underset{ijkl}{\sum }V_{ijkl}{\hat{a}}_{i}^{†}{\hat{a}}_{j}^{†}{\hat{a}}_{l}{\hat{a}}_{k},$$

where $\hat{a}$ and ${\hat{a}}^{†}$ are the annihilation and creation operator, respectively, $h_{ij}=\int dx\phi_{i}^{*}\left(x\right)h\left(r\right)\phi_{j}\left(x\right)$ are the matrix elements of the one-particle noninteracting Hamiltonian *h*(**r**) = − ∇^2^/2 + *v*_ext_(**r**), with *v*_ext_ an external potential, and $V_{ijkl}=\int dxdx^{\prime }\phi_{i}^{*}\left(x\right)\phi_{j}^{*}\left(x^{\prime }\right)v_{c}\left(r,r^{\prime }\right)\phi_{k}\left(x\right)\phi_{l}\left(x^{\prime }\right)$ are the matrix elements of the Coulomb interaction *v*_*c*_. Here **x** = (**r**, *α*) combines space and spin variables and *i*, *j*, … denote both space and spin labels (they will be made explicit only when necessary).

### 2.1 Key Equations of the MEET

Within the MEET the time-ordered 1-body Green’s function *G*(*ω*) at zero temperature is split into removal (*R*) and addition (*A*) parts as *G*(*ω*) = *G* ^*R*^(*ω*) + *G* ^*A*^(*ω*). In the following we concentrate on the diagonal elements of *G*, which are related to photoemission spectra. Within the MEET the diagonal matrix elements of *G* ^*R*/*A*^(*ω*) are written in terms of an effective energy $\delta_{i}^{R/A}\left(\omega \right)$ as (Di Sabatino et al., 2016):

$$\begin{array}{ccc} G_{ii}^{R}\left(\omega \right) & = & \frac{\gamma_{ii}}{\omega -\delta_{i}^{R}\left(\omega \right)-\text{i}\eta }, \end{array}$$ (1)

$$\begin{array}{ccc} G_{ii}^{A}\left(\omega \right) & = & \frac{1-\gamma_{ii}}{\omega -\delta_{i}^{A}\left(\omega \right)+\text{i}\eta }, \end{array}$$ (2)

with *γ*_*ii*_ the diagonal matrix element of the one-body density matrix in a given basis set. We note that a similar effective energy can be introduced also for the off-diagonal elements of *G* ^*R*/*A*^. The spectral function is hence expressed as

$$A_{ii}\left(\omega \right)=\gamma_{ii}\delta \left(\omega -\delta_{i}^{R}\left(\omega \right)\right)+\left(1-\gamma_{ii}\right)\delta \left(\omega -\delta_{i}^{A}\left(\omega \right)\right),$$ (3)

where the symbol *δ* on the right-hand side indicates the Dirac delta function. In our previous works we have chosen the basis set of natural orbitals, i.e., the orbitals which diagonalize the one-body reduced density matrix. In this case *γ*_*ii*_ = *n*_*i*_, i.e., the natural occupation numbers. This choice has been made based on our results on exactly solvable Hubbard clusters, where the MEET performs very well. (Di Sabatino et al., 2016) However this does not guarantee that it remains the best choice for more realistic systems. In fact this is not the case as we shall see.

The effective energy $\delta_{i}^{R/A}\left(\omega \right)$ can be written as an expansion in terms of reduced density matrices. The expression truncated at the level of the one- and two-body reduced density matrices (2-RDM) reads (in the basis of natural orbitals).

$$\begin{array}{ccc} \delta_{i}^{R,\left(1\right)} & = & h_{ii}+\frac{1}{n_{i}}\underset{klm}{\sum }V_{imkl}\Gamma_{klmi}^{\left(2\right)} \end{array}$$ (4)

$$\begin{array}{ccc} \delta_{i}^{A,\left(1\right)} & = & h_{ii}+\frac{1}{\left(1-n_{i}\right)}\underset{k}{\sum }\left(V_{ikik}-V_{ikki}\right)n_{k}\\ & & -\frac{1}{\left(1-n_{i}\right)}\underset{klm}{\sum }V_{imkl}\Gamma_{klmi}^{\left(2\right)}, \end{array}$$ (5)

where $\Gamma_{klmi}^{\left(2\right)}=\left\langle \Psi_{0}^{N}|{\hat{a}}_{i}^{†}{\hat{a}}_{m}^{†}{\hat{a}}_{l}{\hat{a}}_{k}|\Psi_{0}^{N}\right\rangle$ are the matrix elements of the two-body reduced density matrix, with $\Psi_{0}^{N}$ the ground-state wavefunction of the *N*-electron system. As discussed in Ref. (Di Sabatino et al., 2016) the various approximations $\delta_{i}^{R/A,\left(n\right)}\left(\omega \right)$ are related to the *n*-th moments

$$\mu_{n,i}^{R/A}=\frac{\sum_{k}B_{ii}^{k,R/A}{\left(\epsilon_{k}^{R/A}\right)}^{n}}{\sum_{k}B_{ii}^{k,R/A}}$$

of the $G_{ii}^{R/A}\left(\omega \right)$ . Here $\epsilon_{k}^{R}=\left(E_{0}^{N}-E_{k}^{N-1}\right)$ and $\epsilon_{k}^{A}=\left(E_{k}^{N+1}-E_{0}^{N}\right)$ are removal and addition energies, respectively, and

$$\begin{array}{ll} B_{ii}^{k,R} & =\langle \Psi_{0}^{N}|{\hat{c}}_{i}^{†}|\Psi_{k}^{N-1}\rangle \langle \Psi_{k}^{N-1}|{\hat{c}}_{i}|\Psi_{0}^{N}\rangle \\ B_{ii}^{k,A} & =\langle \Psi_{0}^{N}|{\hat{c}}_{i}|\Psi_{k}^{N+1}\rangle \langle \Psi_{k}^{N+1}|{\hat{c}}_{i}^{†}|\Psi_{0}^{N}\rangle , \end{array}$$

with $E_{0}^{N}$ and $\Psi_{0}^{N}$ the ground-state energy and wave function of the *N*-electron system and $E_{k}^{N\pm 1}$ and $\Psi_{k}^{N\pm 1}$ the *k*th state energy and wave function of the (*N* ± 1)-electron system. This allows for a more compact expression of $G_{ii}^{R/A}\left(\omega \right)$ as a continued fraction of moments

$$G_{ii}^{R}=\frac{n_{i}}{\omega -\mu_{1,i}^{R}\frac{\omega -\mu_{1,i}^{R}\ldots }{\omega -\frac{\mu_{2,i}^{R}}{\mu_{1,i}^{R}}\ldots }},$$ (6)

(and similarly for $G_{ii}^{A}$ ). More details on the continued fraction expression for *G* can be found in Refs (Di Sabatino et al., 2016; Di Sabatino et al., 2019). At the level of *δ* ^*R*/*A*,(1)^, the Green’s function depends only on the first moment, while neglecting all the higher-order frequency-dependent corrections. As shown in Ref. (Di Sabatino et al., 2019) this means that each component $G_{ii}^{R/A}$ has only one pole which is a weighted average of all the poles of $G_{ii}^{R/A}$ . If each component of *G* has a predominant quasiparticle peak, this is a good approximation, provided that the approximation to the first moment is accurate enough. At the level of *δ* ^*R*/*A*,(2)^ the Green’s function depends on the first and second moments; since now the corrections are frequency-dependent more poles appear (namely, two removal and two addition poles for each component of *G*, which are visible if the corresponding weights are nonzero). This approximation tends to reproduce the two most dominant removal/addition peaks for each component of *G*. Higher-order moments will produce more poles; however, approximations become quickly uncontrolled (Di Sabatino, 2016), which can lead to unphysical results.

### 2.2 Key Equations of the EKT

Within the EKT one starts from the following approximation for the removal energy $\epsilon_{i}^{R}$ (Kent et al., 1998)

$$\epsilon_{i}^{R}=-\frac{\langle \Psi_{0}^{N}|{\hat{O}}_{i}^{†}\left[\hat{H},{\hat{O}}_{i}\right]|\Psi_{0}^{N}\rangle }{\langle \Psi_{0}^{N}|{\hat{O}}_{i}^{†}{\hat{O}}_{i}|\Psi_{0}^{N}\rangle }$$ (7)

with $\Psi_{0}^{N}$ the ground-state many-body wave function of the *N*-electron system, and ${\hat{O}}_{i}=\sum_{k}C_{ki}^{R}{\hat{a}}_{k}$ , ${\hat{O}}_{i}^{†}=\sum_{k}C_{ki}^{R\;*}{\hat{a}}_{k}^{†}$ , with $\left\{C_{ki}^{R}\right\}$ a set of coefficients to be determined. The stationary condition (with respect to the coefficients $C_{ki}^{R}$ ) for $\epsilon_{i}^{R}$ leads to the secular equation

$$\left(V^{R}-\epsilon_{i}^{R}S^{R}\right)C_{i}^{R}=0,$$ (8)

with $V_{ij}^{R}=-\left\langle \Psi_{0}^{N}|{\hat{a}}_{j}^{†}\left[\hat{H},{\hat{a}}_{i}\right]|\Psi_{0}^{N}\right\rangle$ and *S* ^*R*^ the one-body density matrix $S_{ij}^{R}=\gamma_{ij}=\left\langle \Psi_{0}^{N}|{\hat{a}}_{j}^{†}{\hat{a}}_{i}|\Psi_{0}^{N}\right\rangle$ . If one defines the matrix $\Lambda^{R}={\left[S^{R}\right]}^{-1}V^{R}$ in the basis of natural orbitals, with $S_{ij}^{R}=n_{i}\delta_{ij}$ and works out the commutator in $V_{ij}^{R}$ , one arrives at

$$\Lambda_{ij}^{R}=\frac{1}{n_{i}}\left[n_{i}h_{ji}+\underset{klm}{\sum }V_{jmkl}\Gamma_{klmi}^{\left(2\right)}\right].$$ (9)

The eigenvalues of Λ^*R*^ are the removal energies. (Morrell et al., 1975; Pernal and Cioslowski, 2005) By comparing to Eq. 4 it becomes clear that the diagonal element of Λ^*R*^ are the removal energy of the MEET within the low-order approximation. The diagonal element of Λ^*R*^ are also referred in literature as the energies of the EKT within the diagonal approximation (DEKT).

Similar equations hold for the addition energies. One can indeed define the addition energy $\epsilon_{i}^{A}$ as

$$\epsilon_{i}^{A}=\frac{\langle \Psi_{0}^{N}|\left[\hat{H},{\hat{O}}_{i}\right]{\hat{O}}_{i}^{†}|\Psi_{0}^{N}\rangle }{\langle \Psi_{0}^{N}|{\hat{O}}_{i}{\hat{O}}_{i}^{†}|\Psi_{0}^{N}\rangle }$$ (10)

and in a similar way as for $\epsilon_{i}^{R}$ we arrive at the eigenvalue equation

$$\left(V^{A}-\epsilon_{i}^{A}S^{A}\right)C_{i}^{A}=0,$$ (11)

with $V_{ij}^{A}=\left\langle \Psi_{0}^{N}|{\hat{a}}_{i}\left[\hat{H},{\hat{a}}_{j}^{†}\right]|\Psi_{0}^{N}\right\rangle$ and *S* ^*A*^ related to the one-body density matrix as $S_{ij}^{A}=1-\gamma_{ij}$ . Similarly to the removal energy problem, using the basis of natural orbitals, one can work out the commutator in $V_{ij}^{A}$ and reformulate the problem in terms of the matrix $\Lambda^{A}={\left[S^{A}\right]}^{-1}V^{A}$ ^1^, which reads

$$\begin{array}{ccc} \Lambda_{ij}^{A} & = & \frac{1}{\left(1-n_{i}\right)}\times \\ & & \left[\left(1-n_{i}\right)h_{ji}+\underset{k}{\sum }\left(V_{jkik}-V_{jkki}\right)n_{k}-\underset{klm}{\sum }V_{jmkl}\Gamma_{klmi}^{\left(2\right)}\right]. \end{array}$$ (12)

Again, the diagonal elements of Λ^*A*^ are the MEET addition energies within the approximation given in Eq. 5.^2^

## 3 Mutual Insights and Illustration

Now that we have established the link between the EKT and the MEET we will study how these theories can benefit from mutual insight.

### 3.1 Hubbard Dimer

We use a modified version of the Hubbard dimer in which the on-site Coulomb interaction is different for the two sites. Its Hamiltonian is given by

$$H=-t\underset{\begin{array}{c} i,j=1,2\\ i\neq j \end{array}}{\sum }\underset{\sigma }{\sum }{\hat{a}}_{i\sigma }^{†}{\hat{a}}_{j\sigma }+U_{1}{\hat{n}}_{1↑}{\hat{n}}_{1↓}+U_{2}{\hat{n}}_{2↑}{\hat{n}}_{2↓},$$ (13)

where *i*, *j* run over the sites, ${\hat{n}}_{i\sigma }={\hat{a}}_{i\sigma }^{†}{\hat{a}}_{i\sigma }$ , *U*_*i*_ is the on-site interaction at site *i*, − *t* is the hopping kinetic energy (the site energy *ϵ*_0_ has been set to zero). Contrary to the standard dimer with a unique on-site interaction, in the case of two different on-site interactions the Λ^*R*/*A*^ are not diagonal in the basis of natural orbitals. Therefore, this model allows us to study the effect of the diagonalization on the removal/addition energies in the diagonal approximation. The model can represent the case of a heteronuclear diatomic molecule in a minimal basis set in which the valence orbitals of the two atoms are of different nature, such as HCl or NiO, for example. We note that also using the asymmetric Hubbard dimer with two different site energies the EKT equations are not diagonal in the basis of natural orbitals, however the difference between EKT and DEKT energies is not significant.

#### 3.1.1 Insights Into the EKT

Making the parallel with the MEET, one can readily define the EKT spectral function as.

$$\begin{array}{ll} A_{ii}^{R}\left(\omega \right) & =\gamma_{ii}\delta \left(\omega -\epsilon_{i}^{EKT,R}\right), \end{array}$$ (14)

$$\begin{array}{ll} A_{ii}^{A}\left(\omega \right) & =\left(1-\gamma_{ii}\right)\delta \left(\omega -\epsilon_{i}^{EKT,A}\right), \end{array}$$ (15)

with *γ*_*ii*_ and 1 − *γ*_*ii*_ the diagonal matrix element of the one-body density matrix in the basis which diagonalizes $\Lambda_{ij}^{R}$ and $\Lambda_{ij}^{A}$ , respectively (not necessarily the same for $\Lambda_{ij}^{R}$ and $\Lambda_{ij}^{A}$ ). We notice that the factor *γ*_*ii*_ should refer to a proper one-body density matrix, i.e., a one-body density matrix which fulfils the ensemble *N*-representability constraints. In our case this is guaranteed by the total energy minimization (which includes the constraint 0 ≤ *n*_*i*_ ≤ 1) in RDMFT. Moreover, as for the MEET removal (addition) energies (in its lowest-order approximation), the removal (addition) EKT energies can be interpreted in terms of the first moment of $G_{ii}^{R}$ ( $G_{ii}^{A}$ ), i.e., as weighted averages of all the poles of $G_{ii}^{R}$ ( $G_{ii}^{A}$ ) within the basis that diagonalizes the Λ^*R*^ (Λ^*A*^) matrix. Indeed, inserting a complete set of eigenstates of the (*N* − 1)-electron system in Eq. 7, the commutator can be rewritten as

$$\begin{array}{ccc} \epsilon_{i}^{R} & = & -\underset{k}{\sum }\frac{\langle \Psi_{0}^{N}|{\hat{O}}_{i}^{†}|\Psi_{k}^{N-1}\rangle \langle \Psi_{k}^{N-1}|\left[\hat{H},{\hat{O}}_{i}\right]|\Psi_{0}^{N}\rangle }{\langle \Psi_{0}^{N}|{\hat{O}}_{i}^{†}{\hat{O}}_{i}|\Psi_{0}^{N}\rangle }\\ & = & -\underset{k}{\sum }\frac{\langle \Psi_{0}^{N}|{\hat{O}}_{i}^{†}|\Psi_{k}^{N-1}\rangle \langle \Psi_{k}^{N-1}|{\hat{O}}_{i}|\Psi_{0}^{N}\rangle }{\langle \Psi_{0}^{N}|{\hat{O}}_{i}^{†}{\hat{O}}_{i}|\Psi_{0}^{N}\rangle }\left(E_{k}^{N-1}-E_{0}^{N}\right)\\ & = & \frac{\sum_{k}B_{ii}^{k,R}\epsilon_{k}^{R}}{\sum_{k}B_{ii}^{k,R}}, \end{array}$$ (16)

which is a weighted average of the poles of $G_{ii}^{R}$ . Inserting a complete set of eigenstates of the (*N* + 1)-electron system in Eq. 10 one can show in a similar way that the $\epsilon_{i}^{A}=\sum_{k}B_{ii}^{k,A}\epsilon_{k}^{A}/\sum_{k}B_{ii}^{k,A}$ within the EKT basis. This means that if there are not satellites in the EKT basis set, then the EKT removal/addition energies are exact, provided that one uses the exact first moment.

We notice that very recently Lee *et al.* (Lee et al., 2021), have also proposed an expression for the spectral function from the EKT.

#### 3.1.2 Insights Into the MEET

**FIGURE 1:**
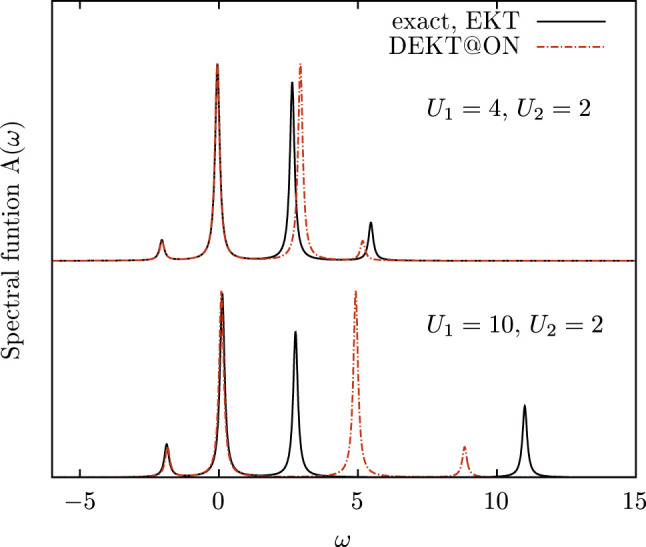
Spectral function of the Hubbard dimer with two different on-site interactions *U*_1_ and *U*_2_ for site 1 and site 2, respectively: exact solution (black curves) *vs.* EKT (black curves, EKT is exact in this case, see text) and DEKT/MEET in the basis of natural orbitals (red curves).

Several choices for an optimal basis set for the MEET expressions are now possible. In previous works we considered the basis of natural orbitals as optimal basis set for the MEET based on the following findings (Di Sabatino, 2016): i) the MEET (in this basis of natural orbitals) gives the exact spectral function at all level of approximations for the symmetric Hubbard dimer using exact density matrices; ii) the MEET in its lowest level of approximation in terms of one- and two-body density matrices gives good results for the spectral function of the (symmetric) Hubbard model with more sites using approximate density matrices. Moreover, for these (symmetric) model systems the Λ^*R*/*A*^ matrices of the EKT are diagonal in the basis of natural orbitals, therefore there is not another better option. For the asymmetric Hubbard dimer instead, and in general for realistic systems, the basis of natural orbitals does not diagonalize the Λ^*R*/*A*^ matrices, therefore the set which diagonalizes these matrices can be a better option for the MEET. We notice that this choice of the optimal basis set can be generally applied to other methods which express *G* as a continued fraction, such as the Lanczos method (Balzer et al., 2011), in order to have more accurate results at a given order of truncation of the series. For example in Figure 1 we report the spectral function of the Hubbard dimer governed by the Hamiltonian in Eq. 13 for two different values of |*U*_1_ − *U*_2_|. The (D)EKT results are obtained using exact density matrices. The results show that the basis which diagonalizes the Λ^*R*/*A*^ matrices is a much better choice than the basis of natural orbitals the more the difference |*U*_1_ − *U*_2_| is large. We also observe that the removal part is less affected by the diagonal approximation than the addition part, and we observe this trend also in more complex systems. The diagonal approximation has been addressed in literature also for realistic systems. (Piris et al., 2013; Kent et al., 1998) In particular in bulk silicon QMC results show that the DEKT slightly overestimates the EKT band gaps. Below we will address this system in more details.

### 3.2 Realistic Systems: The Example of Bulk Si

As an example of realistic systems we use bulk silicon, for which results using the EKT within QMC are reported in Ref. (Kent et al., 1998). The diagonal approximation to the EKT within QMC works very well for the valence states and slightly less well for the conduction states, with a band gap at the Γ point of 4.4 eV *vs.* 3.8 eV from the full EKT compared to 3.4 eV in experiment. (Kent et al., 1998) We note that this discrepency is largely due to the energy of the conduction band at Γ. However, bulk silicon is a relatively weakly correlated system, with hence a predominant quasiparticle-like spectral function for which the EKT is a good approximation. Larger overestimation of the band gap can be expected for strongly correlated systems. This can be understood from the interpretation of the EKT energies as first moments of the one-body Green’s function. However, an important point to stress is that even for these systems, which are a challenge for state-of-the-art *ab initio* methods, such as GW, the EKT would open a gap, in accordance with experiment.

As pointed out in Ref. (Kent et al., 1998) the choice of the trial wave function for QMC calculations is of critical importance. Indeed, as a result of the fixed-node approximation, QMC calculations of the matrix elements of the density matrix and operators **V** ^*R*,*A*^ (see EKT Equations 8, 11) are expected to critically depend on the nodal structure of the trial wave function employed. For the weakly correlated bulk silicon the accurate QMC value of 3.8 eV reported above has been obtained by (Kent et al., 1998) using a standard Slater-Jastrow trial wave function whose nodes are those of a single determinant consisting of LDA orbitals. For more strongly correlated systems the wave function acquires a significant multi-determinant character and getting physically meaningful nodes becomes much more difficult. It is thus useful to use the EKT within alternative approaches.

**FIGURE 2:**
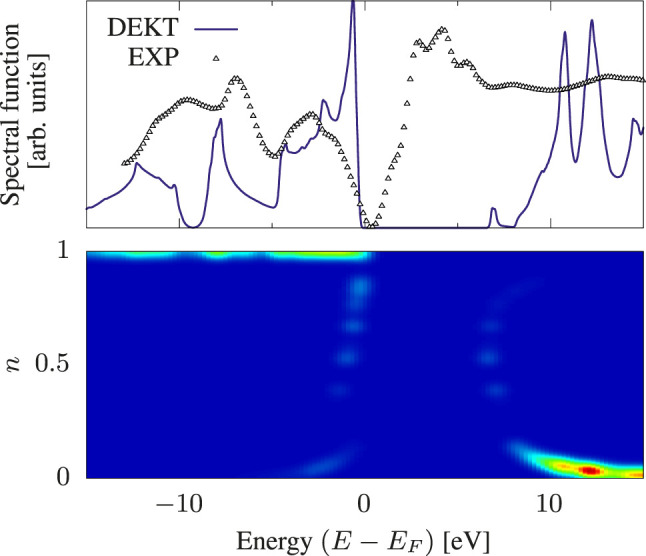
Spectral function of bulk Si within DEKT (violet solid line). The experimental photoemission spectrum (small triangles) is taken from Ref. (Chelikowsky et al., 1989). The color map illustrates the occupation numbers *n* *_i_* that play a role into the spectrum for the reported energy range.

**FIGURE 3:**
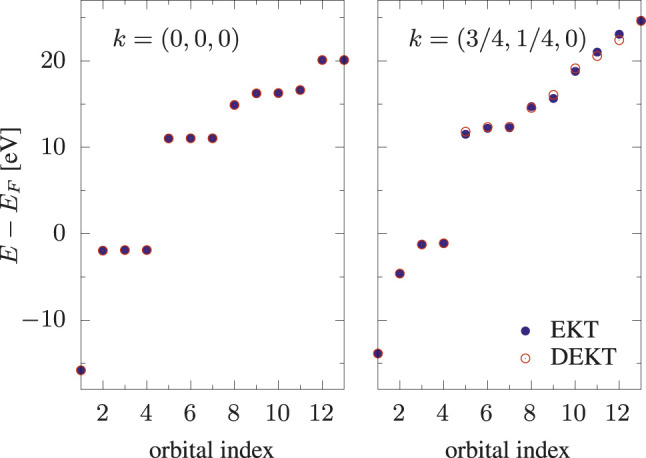
Removal/addition energies of bulk Si: EKT (filled circles) *vs* DEKT (empty circles) for two *k*-points, *k* = (0, 0, 0) and *k* = (3/4, 1/4, 0).

In our previous works (Di Sabatino et al., 2016, 2019, 2021) we used reduced-density matrix functional theory (RDMFT) (Gilbert, 1975) to find approximations to the one- and two-body density matrices which are needed in the MEET equations. More specifically the two-body density matrix in the MEET equations is approximated using the Power functional, which is given by $\Gamma_{ijkl}^{\left(2\right)}=n_{i}n_{j}\delta_{il}\delta_{jk}-n_{i}^{\alpha }n_{j}^{\alpha }\delta_{ik}\delta_{jl}$ (*α* = 0.65). (Sharma et al., 2013) The optimal natural orbitals {*ϕ*_*i*_} and occupation numbers {*n*_*i*_} are obtained by minimizing the total energy which is expressed in terms of *γ* and Γ^(2)^, with Γ^(2)^ as functional of *γ*. The Power functional is used also to approximate Γ^(2)^[*γ*] in the energy functional. In this work we use the same protocol for the EKT equations. We implemented the EKT equations in a modified version of the full-potential linearized augmented plane wave (FP-LAPW) code ELK (Elk, 2004), with practical details of the calculations following the scheme described in Ref. (Sharma et al., 2008). For bulk Si we used a lattice constant of 5.43 Å  and a Γ-centered 8 × 8 × 8 **k**-point sampling of the Brillouin zone. In Figure 2 we report the DEKT spectral function of bulk silicon: the direct band gap at Γ is 12.9 eV, while the fundamental band gap is 8.18 eV, which is larger than the experimental one of 1.12 eV (Sze, 1969). We also observe a spurious peak in the band gap due to the fact that the Power functional produces occupation numbers which strongly deviate from 1 and 0 (as one would expect for this weakly correlated system) close to the Fermi energy (see bottom panel of Figure 2). This is in contrast with the QMC results. Note that we observe similar deviations from 1 and 0 also for other weakly correlated systems, such as diamond, which points to a problem of the Power functional for the description of occupation numbers of weakly correlated systems. Moreover, the full EKT does not show any improvement over the DEKT, as one can see from Figure 3, in which the EKT and DEKT energies are reported: the fundamental band gap is reduced by only 0.06 eV. This is again in contrast with the QMC results in which, although small, there is a significant difference. We attribute this different trend to the use of the Power functional, which contracts the four-point 2-RDM to two points only, and hence probably mitigating the impact of the diagonalization of the Λ^*R*/*A*^ matrices. These results on bulk Si indicate that, although the EKT/DEKT are expected to overestimate the band gap (even using very accurate density matrices, as for example shown in the case of the Hubbard model (Di Sabatino et al., 2016)), this overestimation can be much amplified by using approximations such as the Power functional. More advanced approximations to Γ^(2)^ are hence needed, which give, in particular, more accurate natural occupation numbers. We notice that varying *α* would change the band gap width. In particular *α* = 1 would give the HF band gap, which still overestimates the experimental one, whereas decreasing *α* would increase the overestimation of the band gap.

## 4 Conclusions and Perspectives

We linked our recently derived Many-Body Effective Energy Theory (MEET) for the calculation of photoemission spectra to the Extended Koopman’s Theorem (EKT). Within the lowest level of approximation in terms of one- and two-body density matrices, the MEET equations correspond to the so-called diagonal approximation to the EKT (DEKT) equations. This allowed us to readily extend the EKT to the calculation of an approximate spectral function as well as to give an alternative interpretation of the EKT in terms of moments of the one-body Green’s function. Using the test case of the Hubbard dimer with two different on-site interactions *U*_1_ and *U*_2_ for site 1 and site 2 we showed the effect of the basis set on the MEET (removal and addition) energies: in particular HOMO-LUMO gap in the basis sets which solve the EKT secular equations (one basis set for the valence part and one for the conduction part) is smaller than the HOMO-LUMO gap obtained using the natural orbital basis set. These results are in line with the EKT results reported in literature for bulk Si using QMC. We have implemented the EKT within reduced-density matrix functional theory (RDMFT), which offers a convenient computationally affordable framework to treat extended systems. However one has to rely on approximate one- and two-body density matrices. We showed that using the currently available approximations the DEKT band gap of Si largely deviates from the DEKT value obtained using QMC (12.9 eV *vs* 4.4 eV at the Γ point) and, moreover, there is no effect of the basis set (EKT *vs* DEKT) on the DEKT energies, contrary to what is observed within QMC, where, although small, there is a significant difference. These results on bulk Si indicate that, although the EKT/DEKT are expected to overestimate the band gap (even using very accurate density matrices), this overestimation can be much amplified by commonly used approximations in RDMFT. This also explains the huge overestimation of the band gap obtained by the MEET within RDMFT in strongly correlated systems such as paramagnetic NiO. We are currently working on improving approximations to correlation in RDMFT by introducing some form of screening (for example the screening due to electron-hole excitations as in GW), which is of particular importance in solids.

## Data Availability

The original contributions presented in the study are included in the article/Supplementary Material, further inquiries can be directed to the corresponding authors.

## Footnotes

1 Since in the basis of natural orbitals the *S* ^*R*^ (*S* ^*A*^) matrix is a diagonal matrix with the natural occupation numbers *n*_*i*_ (1-*n*_*i*_) as elements, the invertibility of this matrix is strictly related to the non-existence of so-called pinned states, i.e. states with occupation numbers equal to 1 or 0. This is an important question that has several consequences (e.g., Giesbertz and van Leeuwen, 2013; Baldsiefen et al., 2015). Here we assume that *S* ^*R*^ (*S* ^*A*^) is invertible in a restricted space (of natural orbitals) in which the corresponding KS orbitals are occupied (unoccupied). This is a reasonable assumption.

2 Note that the standard EKT equations in RDMFT (Morrell et al., 1975) present a prefactor $1/\sqrt{n_{i}n_{j}}$ ( $1/\sqrt{\left(1-n_{i}\right)\left(1-n_{j}\right)}$ ) instead of 1/*n*_*i*_ (1/(1 − *n*_*i*_)) in Eq. 9 (Eq. 12); these two choices yield the same eigenvalues for the matrix **Λ** ^*R*^ (**Λ** ^*A*^).
